# Outcomes of total versus partial colectomy in fulminant *Clostridium difficile* colitis: a propensity matched analysis

**DOI:** 10.1186/s13017-022-00414-2

**Published:** 2022-02-13

**Authors:** Nasim Ahmed, Yen-Hong Kuo

**Affiliations:** 1 Division of Trauma & Surgical Critical Care, Jersey Shore University Medical Center, 1945 State Route 33, Neptune, NJ 07754 USA; 2 Office of Research Administration, Jersey Shore University Medical Center, Neptune, NJ USA; 3 Hackensack Meridian School of Medicine, Nutley, NJ USA

**Keywords:** Fulminant *Clostridium Difficile* Colitis, Partial colectomy, Total colectomy, Mortality

## Abstract

**Background:**

The Total Abdominal Colectomy (TAC) is the recommended procedure for Fulminant *Clostridium Difficile* Colitis (FCDC), however, occasionally, FCDC is also treated with partial colectomies. The purpose of the study was to identify the outcomes of partial colectomy in FCDC cases.

**Method:**

The National Surgical Quality Improvement Program database was accessed and eligible patients from 2012 through 2016 were reviewed. Patients 18 years and older who were diagnosed with FCDC and who underwent colectomies were included in the study. Patients’ demography, clinical characteristics, comorbidities, mortality, morbidities, length of hospital stay and discharge disposition were compared between the group who underwent partial colectomy and the group who underwent TAC. Univariate analysis followed by propensity matching was performed. A *P* value of < 0.05 is considered as statistically significant.

**Results:**

Out of 491 patients who qualified for the study, 93 (18.9%) patients underwent partial colectomy. The pair matched analysis showed no significant difference in patients’ characteristics and comorbidities in the two groups. There was no significant difference found in mortality between the two groups (30.1% vs. 30.1%, *P* > 0.99). There were no differences found in the median [95% CI] hospital length of stay (LOS) (23 days [19–31] vs. 21 [17–25], *P* = 0.30), post-operative complications (all *P* > 0.05), and discharged disposition to home ( 33.8% vs. 43.1%) or transfer to rehab (21.55 vs. 12.3%, *P* = 0.357) between the TAC and partial colectomy groups.

**Conclusion:**

The overall 30 days mortality remains very high in FCDC. Partial colectomy did not increase risk of mortality or morbidities and LOS.

**Level of evidence:**

Level IV.

**Study type:**

Observational cohort.

## Introduction

Fulminant *Clostridium Difficile* Colitis (FCDC) is one of the severe conditions of colon that is associated with very high mortality [1]. Approximately 4% of patients with *Clostridium difficile* colitis progress into fulminant course [2]. Aggressive resuscitation and early colectomy resulted in lower mortality [3, 4]. Total abdominal colectomy (TAC) is a standardized procedure for FCDC [5].

Alternate to TAC, another procedure was proposed almost 10 years ago, loop ileostomy (LI) and colonic lavage [6]. This new procedure was compared to TAC but no significant difference in overall mortality was found in a small study including 10 patients of (LI) compared to 13 patients of TAC [7].

Recent studies from the NSQIP database showed colectomy is still the procedure of choice [3, 8] but as an alternative to TAC, studies suggest a partial colectomy which does not appear to increase mortality [8]. In addition, the partial colectomy could ensure that part of the colon can be saved thus minimizing the metabolic consequences that ensues with TAC [9]. The study that compared the partial colectomy with standardized total abdominal colectomy for FCDC adjusted the characteristics of patients on multivariable logistic regression analysis [8]. However, propensity score comparison methodology was reported to be a better mode of performing observational studies [10]. As a result, we decided to conduct a study using propensity score matching analysis to find out the outcomes of patients who underwent partial colectomy for FCDC.

## Methods

All adult patients age 18 years and older who were diagnosed with *Clostridium difficile* colitis and underwent emergency colectomy for the indication of *Clostridium difficile* colitis were included in the study. The data came from the National Surgical Quality Improvement Program (NSQIP) database from the calendar years 2012–2016. The American College of Surgeons developed the NSQIP database for the improvement of outcomes in surgical patients [11]. More than 700 institutions across the US participate in the NSQIP. The *Clostridium difficile* colitis defined as if the patient develops diarrhea with positive *Clostridium difficile* on laboratory test of stool by culture or PCR assay or Glutamate dehydrogenase EIA/ latex agglutination or cytotoxin test. Excluded from the study all emergency colectomies that were performed other than the indication of *Clostridium difficile* colitis. All elective colectomies were also excluded from the study.

We analyzed two groups, (partial colectomy [when a segment of the colon {right sided or left sided} was removed] versus total colectomy [where the entire colon was removed]), looking at gender, race, preoperative sepsis status, white blood cell counts, blood transfusion before the surgery and ventilator dependent respiratory failure prior to surgery, the American Society of Anesthesiologists (ASA) classification of the surgery, wound classification and comorbidities; history of diabetes mellitus (DM), smoking, chronic obstructive pulmonary disease (COPD), ascites, hypertension on medication (HTN), congestive heart failure, chronic renal failure (CRF), CRF on dialysis (CRF-D), disseminated cancer, steroid use. All comorbidities, sepsis and septic shock were defined as per NSQIP data dictionary.

The primary outcome of the study was 30 days all-cause mortality, while secondary outcomes were post-operative complications, hospital length of stay and discharge disposition.

### Statistics

First, patient demographic information and outcomes were summarized using summary statistics (median with interquartile range (IQR) [first quartile–third quartile]) for continuous variables, and frequency and percentage for categorical variables). Then, the group that underwent total abdominal colectomy was compared with the group that underwent partial colectomy on patient’s demography, diseases severity, comorbidities, and outcomes. The Wilcoxon Rank Sum test was used for continuous variables, and the Chi-square test was used for the categorical variables.

The propensity score for total abdominal colectomy was calculated for each patient and the one-to-one matching was performed using the “nearest neighbor” as the matching method to pair a subject who had TAC with the subject who underwent partial colectomy. The propensity score matching was performed using the R package “MatchIt” [12]. The propensity scores were calculated using all the variables that may have impacted the decision to perform one procedure type versus other procedure type and that included (gender, age, race [white versus non-white], sepsis status, blood transfusion, respiratory failure, ASA class, wound class and all comorbidities mentioned above. After matching, the numeric and graphical diagnostics were used to evaluate the improvement in the variables. Again, summary statistics were performed as described above. One-to-one comparison between the two matched groups was performed using Wilcoxon Signed Rank test for the continuous variables. The McNemar’s test was used to compare the categorical variables between the two matched groups. If the level of a categorical variable was more than two, the Stuart-Maxwell test was used. For the total hospital length stay, the Kaplan–Meier procedure was used to estimate the median time, and the standard error was estimated using the Greenwood’s formula. The log-rank test was used to compare the time (Kaplan–Meier curves) between the two groups. The 2-sided *P* value was reported for each test. A *P* value of < 0.05 was considered an indication of statistical significance. Statistical analysis was performed using the R language [13].

## Results

### Patients’ characteristics & Univariate analysis

Out of 491 patients who qualified for the study, 398 (81.1 %) patients underwent total abdominal colectomy. Only 93 (18.9%) patients underwent partial colectomy. Fifty one out of 93 (54.83%) patients underwent right sided colectomy and remaining 42 (45.17%) patients had left sided colectomy. Approximately 84% of patient underwent colectomy for toxic colon and approximately 16% of patients underwent colectomy for perforation. The median [IQR] age of the patient who underwent partial colectomy was 66 [55–75], the male and female distribution was almost split equally with slight increase of male dominance, ~ 53% and about 77% of patients were Caucasians. There were significant differences found between the two groups, TAC and partial colectomy groups, regarding the presence of septic shock prior to surgery (67.8% versus 52.7%, *P* = 0.03). TAC group presented with higher percentage of life threatening of ASA class (66.3% versus 59.1%, *P* = 0.029) and found to have higher percentage of patients with history of steroid use (22.9% versus 12.9%, *P* = 0.047). Significantly higher proportion of patients in TAC group mounted severe leukocytosis (≥ 20 × 10^9^/L) (Table 1).

**Table 1:** Comparison of characteristics of patients between the two groups (TAC versus partial colectomy) before propensity matching

Variable	All Patients N = 491	Total Abdominal Colectomy N = 398	Partial Colectomy N = 93	*P* value
Age (years), Median [Q1-Q3]	67 [57–76]	67 [58–75]	66 [55–75]	0.31
Gender				0.22
Female	263 (53.6)	219 (55)	44 (47.3)	
Male	228 (46.4)	179 (45)	49 (52.7)	
Admitted from				0.502
From acute care hospital inpatient	95 (19.3)	82 (20.6)	13 (14)	
Not transferred (admitted from home)	275 (56)	215 (54)	60 (64.5)	
Nursing home—Chronic care—Intermediate care	75 (15.3)	61 (15.3)	14 (15.1)	
Outside emergency department	38 (7.7)	33 (8.3)	5 (5.4)	
Transfer from other	6 (1.2)	5 (1.3)	1 (1.1)	
Unknown	2 (0.4)	2 (0.5)	0 (0)	
Race, white, *n* (%)	341 (69.5)	269 (67.6)	72 (77.4)	0.084
Sepsis status, *n* (%)				0.031
None	35 (7.1)	28 (7)	7 (7.5)	
Sepsis	111 (22.6)	80 (20.1)	31 (33.3)	
Septic Shock	319 (65)	270 (67.8)	49 (52.7)	
SIRS	26 (5.3)	20 (5)	6 (6.5)	
WBC count, 10^9^/L, *n* (%)				< 0.001
4–11.9	97 (19.8)	72 (18.1)	25 (26.9)	
12–19.9	85 (17.3)	57 (14.4)	28 (30.1)	
20–34.9	103 (21)	89 (22.4)	14 (15.1)	
35–44.9	56 (11.4)	52 (13.1)	4 (4.3)	
≥ 45	51 (10.4)	41 (10.3)	10 (10.8)	
< 4	34 (6.9)	24 (6)	10 (10.8)	
Unknown	64 (13.1)	62 (15.6)	2 (2.2)	
Diabetes, *n* (%)				0.547
INSULIN	64 (13)	51 (12.8)	13 (14)	
NO	387 (78.8)	312 (78.4)	75 (80.6)	
NON-INSULIN	40 (8.1)	35 (8.8)	5 (5.4)	
Smoking, *n* (%)				0.724
No	379 (77.2)	309 (77.6)	70 (75.3)	
Yes	112 (22.8)	89 (22.4)	23 (24.7)	
Ventilator dependency^a^, *n* (%)				0.384
No	327 (66.6)	261 (65.6)	66 (71)	
Yes	164 (33.4)	137 (34.4)	27 (29)	
COPD, *n* (%)				0.387
No	371 (75.6)	297 (74.6)	74 (79.6)	
Yes	120 (24.4)	101 (25.4)	19 (20.4)	
Ascites, *n* (%)				0.985
No	430 (87.6)	348 (87.4)	82 (88.2)	
Yes	61 (12.4)	50 (12.6)	11 (11.8)	
CHF, *n* (%)				0.764
No	442 (90)	357 (89.7)	85 (91.4)	
Yes	49 (10)	41 (10.3)	8 (8.6)	
HTN, *n* (%)				0.721
No	190 (38.7)	152 (38.2)	38 (40.9)	
Yes	301 (61.3)	246 (61.8)	55 (59.1)	
CRF, *n* (%)				0.226
No	398 (81.1)	318 (79.9)	80 (86)	
Yes	93 (18.9)	80 (20.1)	13 (14)	
CRF on dialysis, *n* (%)				0.85
No	422 (85.9)	341 (85.7)	81 (87.1)	
Yes	69 (14.1)	57 (14.3)	12 (12.9)	
Disseminated cancer, *n* (%)				0.002
No	452 (92.1)	374 (94)	78 (83.9)	
Yes	39 (7.9)	24 (6)	15 (16.1)	
Steroid, *n* (%)				0.047
No	388 (79)	307 (77.1)	81 (87.1)	
Yes	103 (21)	91 (22.9)	12 (12.9)	
Weight loss				0.825
No	454 (92.5)	367 (92.2)	87 (93.5)	
Yes	37 (7.5)	31 (7.8)	6 (6.5)	
Coagulopathy, *n* (%)				0.57
No	388 (79)	312 (78.4)	76 (81.7)	
Yes	103 (21)	86 (21.6)	17 (18.3)	
Blood transfusion^a^, *n* (%)				0.525
No	437 (89)	352 (88.4)	85 (91.4)	
Yes	54 (11)	46 (11.6)	8 (8.6)	
Wound class, *n* (%)				< 0.001
1-Clean	3 (0.6)	1 (0.3)	2 (2.2)	
2-Clean/Contaminated	91 (18.5)	82 (20.6)	9 (9.7)	
3-Contaminated	166 (33.8)	141 (35.4)	25 (26.9)	
4-Dirty/Infected	231 (47)	174 (43.7)	57 (61.3)	
ASA class, *n* (%)				0.029
2-Mild Disturb	6 (1.2)	3 (0.8)	3 (3.2)	
3-Severe Disturb	75 (15.3)	56 (14.1)	19 (20.4)	
4-Life Threat	319 (65)	264 (66.3)	55 (59.1)	
5-Moribund	90 (18.3)	75 (18.8)	15 (16.1)	
None assigned	1 (0.2)	0 (0)	1 (1.1)	

ASA; American Society of Anesthesiology, CHF; Congestive heart failure, CRF; chronic renal failure,

COPD; chronic obstructive pulmonary disease, Q1-Q3 ; first quartile-third quartile, interquartile range (IQR), SIRS; systemic inflammatory response syndrome. WBCs; White blood cell counts

^a^Prior to surgery

n; number of patients, %; percentage

### Propensity matching analysis

The propensity matching created 93 pairs. There was significant improvement in patients’ characteristics after the matching. The pair matched analysis showed that all the differences between the two groups found in univariate analysis were balanced after the matching. (Figure) 1.

**Fig. 1:**
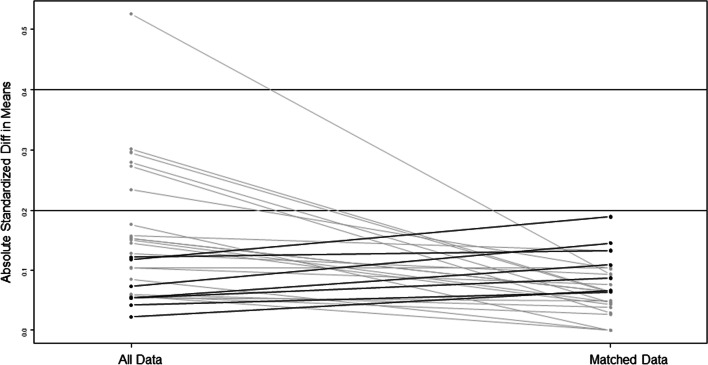
Showing the improvement in standardized mean differences in variables after propensity matching

There were no differences between the groups, TAC versus partial colectomy, regarding median age 65[57–75] vs. 66 [55–75], race [Caucasians] 73.1% vs. 77.4%, gender [male] (49.5% vs. 52.7%), septic shock prior to surgery (55.9% vs. 52.7%) and ventilator dependent respiratory failure (37.6% vs. 29%) and comorbidities, all *P* values were > 0.05 (Table 2).

**Table 2:** Comparison of characteristics of patients between the two groups (TAC versus partial colectomy) after propensity matching

Variable	All Patients, n = 186	Total Abdominal Colectomy, n = 93	Partial Colectomy, n = 93	P-Value
Age (years), Median [Q1-Q3]		65 [57–75]	66 [55 –75]	0.71
Gender				0.755
Female	91 (48.9)	47 (50.5)	44 (47.3)	
Male	95 (51.1)	46 (49.5)	49 (52.7)	
Admitted from				NA
From acute care hospital inpatient	33 (17.7)	20 (21.5)	13 (14)	
Not transferred (admitted from home)	111 (59.7)	51 (54.8)	60 (64.5)	
Nursing home—Chronic care—Intermediate care	25 (13.4)	11 (11.8)	14 (15.1)	
Outside emergency department	10 (5.4)	5 (5.4)	5 (5.4)	
Transfer from other	5 (2.7)	4 (4.3)	1 (1.1)	
Unknown	2 (1.1)	2 (2.2)	0 (0)	
Race, white, *n* (%)	140 (75.3)	68 (73.1)	72 (77.4)	0.596
Sepsis status^a^, *n* (%)				0.956
None	13 (7)	6 (6.5)	7 (7.5)	
Sepsis	60 (32.3)	29 (31.2)	31 (33.3)	
Septic Shock	101 (54.3)	52 (55.9)	49 (52.7)	
SIRS	12 (6.5)	6 (6.5)	6 (6.5)	
WBC count, 10^9^/L, *n* (%)				NA
4–11.9	45 (24.2)	20 (21.5)	25 (26.9)	
12–19.9	43 (23.1)	15 (16.1)	28 (30.1)	
20–34.9	37 (19.9)	23 (24.7)	14 (15.1)	
35–44.9	11 (5.9)	7 (7.5)	4 (4.3)	
≥ 45	15 (8.1)	5 (5.4)	10 (10.8)	
< 4	19 (10.2)	9 (9.7)	10 (10.8)	
Unknown	16 (8.6)	14 (15.1)	2 (2.2)	
Diabetes, *n* (%)				0.878
INSULIN	28 (15.1)	15 (16.1)	13 (14)	
NO	149 (80.1)	74 (79.6)	75 (80.6)	
NON-INSULIN	9 (4.8)	4 (4.3)	5 (5.4)	
Smoking, *n* (%)				0.86
No	142 (76.3)	72 (77.4)	70 (75.3)	
Yes	44 (23.7)	21 (22.6)	23 (24.7)	
Ventilator dependency^a^, *n* (%)				0.268
No	124 (66.7)	58 (62.4)	66 (71)	
Yes	62 (33.3)	35 (37.6)	27 (29)	
COPD, *n* (%)				0.458
No	153 (82.3)	79 (84.9)	74 (79.6)	
Yes	33 (17.7)	14 (15.1)	19 (20.4)	
Ascites, *n* (%)				0.831
No	162 (87.1)	80 (86)	82 (88.2)	
Yes	24 (12.9)	13 (14)	11 (11.8)	
CHF, *n* (%)				> 0.99
No	169 (90.9)	84 (90.3)	85 (91.4)	
Yes	17 (9.1)	9 (9.7)	8 (8.6)	
HTN, *n* (%)				0.551
No	71 (38.2)	33 (35.5)	38 (40.9)	
Yes	115 (61.8)	60 (64.5)	55 (59.1)	
CRF, *n* (%)				> 0.99
No	160 (86)	80 (86)	80 (86)	
Yes	26 (14)	13 (14)	13 (14)	
CRF on dialysis, *n* (%)				0.838
No	160 (86)	79 (84.9)	81 (87.1)	
Yes	26 (14)	14 (15.1)	12 (12.9)	
Disseminated Cancer, *n* (%)				> 0.99
No	157 (84.4)	79 (84.9)	78 (83.9)	
Yes	29 (15.6)	14 (15.1)	15 (16.1)	
Steroid, *n* (%)				0.814
No	164 (88.2)	83 (89.2)	81 (87.1)	
Yes	22 (11.8)	10 (10.8)	12 (12.9)	
Weight loss				0.752
No	176 (94.6)	89 (95.7)	87 (93.5)	
Yes	10 (5.4)	4 (4.3)	6 (6.5)	
Coagulopathy, *n* (%)				> 0.99
No	152 (81.7)	76 (81.7)	76 (81.7)	
Yes	34 (18.3)	17 (18.3)	17 (18.3)	
Blood transfusion^a^, *n* (%)				0.789
No	168 (90.3)	83 (89.2)	85 (91.4)	
Yes	18 (9.7)	10 (10.8)	8 (8.6)	
Wound class, *n* (%)				NA
1-Clean	2 (1.1)	0 (0)	2 (2.2)	
2-Clean/Contaminated	29 (15.6)	20 (21.5)	9 (9.7)	
3-Contaminated	56 (30.1)	31 (33.3)	25 (26.9)	
4-Dirty/Infected	99 (53.2)	42 (45.2)	57 (61.3)	
ASA class, *n* (%)				NA
2-Mild Disturb	5 (2.7)	2 (2.2)	3 (3.2)	
3-Severe Disturb	33 (17.7)	14 (15.1)	19 (20.4)	
4-Life Threat	112 (60.2)	57 (61.3)	55 (59.1)	
5-Moribund	35 (18.8)	20 (21.5)	15 (16.1)	
None assigned	1 (0.5)	0 (0)	1 (1.1)	

ASA; American Society of Anesthesiology, CHF; Congestive heart failure, CRF; chronic renal failure,

COPD; chronic obstructive pulmonary disease, Q1-Q3; first quartile-third quartile,  interquartile range (IQR), SIRS; systemic inflammatory response syndrome. WBCs; White blood cell counts

^a^Prior to surgery

n; number of patients, %; percentage

NA; not applicable

There was no significant difference in mortality between the TAC and partial colectomy groups (30.1% vs. 30.1%, *P* > 0.99). The median [95% CI] hospital length of stay between the TAC and partial colectomy was (23 [19–31] vs. 21 [17–25], *P* = 0.30). There was no significant difference found between the groups, TAC and partial colectomy, regarding the discharged disposition to home (33.8% vs. 43.1%) or transfer to rehab (21.55 vs. 12.3%, *P* = 0.357) (Table 3).

**Table 3:** Mortality and hospital length of stay between the two groups, TAC versus partial colectomy in matched data set

Variable	All patients, *n* = 186	Total abdominal colectomy, *n* = 93	Partial colectomy, n = 93	*P* value
Mortality				> 0.99
Survived	130 (69.9)	65 (69.9)	65 (69.9)	
Died	56 (30.1)	28 (30.1)	28 (30.1)	
Hospital length of stay (days), Median [95% CI]		23 [19–31]	21 [17–25]	0.30

There were no significance differences found between the two groups regarding surgical site infections, incidence of pneumonia, urinary tract infections, sepsis, septic shock, return to operating room, failure to wean from the ventilator and readmission rates Table 4.

**Table 4:** Post-operative complications between the groups in matched data

Variable	All patients, *n* = 186	Total abdominal colectomy, *n* = 93	Partial colectomy, *N* = 93	*P* value
Superficial SSI				> 0.99
No	182 (97.8)	91 (97.8)	91 (97.8)	
Yes	4 (2.2)	2 (2.2)	2 (2.2)	
Deep Incisional SSI				NA
Yes	2 (1.1)	0 (0)	2 (2.2)	
No	184 (98.9)	93 (100)	91 (97.8)	
Organ/Space SSI				> 0.99
No	171 (91.9)	85 (91.4)	86 (92.5)	
Yes	15 (8.1)	8 (8.6)	7 (7.5)	
Wound Disruption				0.343
No	176 (94.6)	90 (96.8)	86 (92.5)	
Yes	10 (5.4)	3 (3.2)	7 (7.5)	
Pneumonia				0.176
No	145 (78)	68 (73.1)	77 (82.8)	
Yes	41 (22)	25 (26.9)	16 (17.2)	
Unplanned intubation				> 0.99
No	159 (85.5)	80 (86)	79 (84.9)	
Yes	27 (14.5)	13 (14)	14 (15.1)	
Pulmonary embolism				0.617
No	182 (97.8)	90 (96.8)	92 (98.9)	
Yes	4 (2.2)	3 (3.2)	1 (1.1)	
Ventilator dependency^a^				0.766
No	91 (48.9)	47 (50.5)	44 (47.3)	
Yes	95 (51.1)	46 (49.5)	49 (52.7)	
Acute renal failure				0.814
Yes	18 (9.7)	10 (10.8)	8 (8.6)	
No	168 (90.3)	83 (89.2)	85 (91.4)	
UTI				> 0.99
No	177 (95.2)	89 (95.7)	88 (94.6)	
Yes	9 (4.8)	4 (4.3)	5 (5.4)	
CVA				> 0.99
No	183 (98.4)	92 (98.9)	91 (97.8)	
Yes	3 (1.6)	1 (1.1)	2 (2.2)	
Cardiac arrest requiring CPR				0.096
Yes	17 (9.1)	12 (12.9)	5 (5.4)	
No	169 (90.9)	81 (87.1)	88 (94.6)	
MI				0.371
Yes	5 (2.7)	1 (1.1)	4 (4.3)	
No	181 (97.3)	92 (98.9)	89 (95.7)	
Blood transfusion^a^				0.888
No	82 (44.1)	42 (45.2)	40 (43)	
Yes	104 (55.9)	51 (54.8)	53 (57)	
DVT				NA
Yes	13 (7)	1 (6.5)	7 (7.5)	
No	173 (93)	87 (93.5)	86 (92.5)	
Sepsis^a^				> 0.99
No	168 (90.3)	84 (90.3)	84 (90.3)	
Yes	18 (9.7)	9 (9.7)	9 (9.7)	
Septic shock^a^				0.551
No	109 (58.6)	57 (61.3)	52 (55.9)	
Yes	77 (41.4)	36 (38.7)	41 (44.1)	
Return to OR				> 0.99
No	163 (87.6)	81 (87.1)	82 (88.2)	
Yes	23 (12.4)	12 (12.9)	11 (11.8)	
Readmission				0.169
No	167 (89.8)	87 (93.5)	80 (86)	
Yes	19 (10.2)	6 (6.5)	13 (14)	

SSI, surgical site infection; UTI, urinary tract infection; CVA, cerebrovascular accidents; MI, Myocardial infarction; DVT, deep vein thrombosis

^a^Post-operative

## Discussion

Our study showed that the majority, ~ 81% of FCDC patients underwent total abdominal colectomy while only ~ 19% of patients had partial colectomy. The all-cause 30-days mortality in the matched group was 30.1%. Partial colectomy did not show any difference in overall mortality or post-operative complications and discharge disposition to home.

Prior studies showed that early colectomy had a better survival probability than no colectomy [4, 14]. Total abdominal colectomy has been the practice pattern for many decades in fulminant cases of FCDC [5]. In 2015, World Society of Emergency Surgery (WSES) recommendation was to perform early TAC in the management of FCDC [15]. The updated WSES guidelines in 2019 kept the TAC as a primary choice of surgical intervention [16].

Very few prior studies have examined the comparison of mortality outcome of TAC with partial colectomy. A study examined the surgical mortality of the FCDC found that patients underwent partial colectomy had the worse outcome than the TAC [17]. The major limitation of the study was a very small sample size. The total number of patients included in the study was 14 and the major reasons for the high mortality in partial colectomy were not very clear. Byrn and colleagues examined 73 patients with FCDC who underwent colectomy [18]. Most colectomies (86%) were subtotal colectomy, only 4 patient had right hemicolectomy and 5 had left hemicolectomy and one patient had total colectomy. One patient who had left hemicolectomy was converted to total colectomy. No significant difference was found in overall mortality whether the patient underwent partial colectomy or subtotal colectomy (10% vs 38%, respectively; *P* > 0.05). A recent NSQIP database study included all patients with FCDC who underwent colectomies from 2007 through 2015 [8]. The study consisted of 733 patients and found slightly higher mortality rate in partial colectomy group when compared to TAC (37.1% vs 34.7%, P=0.58) in univariate analysis. However, multiple logistic regression analysis did not show any significant difference in mortality of partial colectomy group when compared with TAC, the odds ratio [OR] was 1.21, 95% CI 0.76 to 1.96.

Contrary to above studies, our study included relatively recent NSQIP data set and used propensity-matched analysis, which is better modality for observational study [10]. Our results showed 81% of patients underwent TAC as recommended by the WSES [16]. Approximately 19% of patients underwent partial colectomy. The reasons for lower compliance with WSES were not available. There is a possibility that in certain cases, the point of care surgeon made the decision to perform partial colectomy was based on findings observed during the operation. Patients who underwent partial colectomy showed no difference in 30 days mortality (30.1% vs. 30.1%) when compared with TAC. Our mortality was little lower than the published report [8]. The reason may be that we used the most recent dataset that may have reflected the better selection of patients to critical care management and aggressive treatment of the FCDC [19]. The other reason for lower mortality in our study could be the inclusion of all comorbidities in our propensity-matching model that can influence the post-operative mortality [20]. Our study did not find any significant difference in median hospital length of stay and 30-day post-operative complications regardless of the type of surgery was performed (TAC vs. partial colectomy). Our study added one more outcome to evaluate the discharged disposition to home and found no significant difference between the TAC vs. partial colectomy Table 3.

### Limitation

The study was done from the NSQIP database; however, the database lacks the detailed information of the some of the patients’ characteristics, the timing of the contraction of the clostridium difficile colitis, progression to FCDC and the timing of the colectomy from the time of identification of the FCDC. We used the most recommended analysis method of observational study, the propensity score matching. However, that method does not take into account any unobserved or unmeasured variables that may have influenced the results.

## Conclusion

The surgical mortality of FCDC remains high. Total abdominal colectomy was the procedure of choice and adapted by majority of surgeons. Partial colectomy did not increase the risk of 30 days mortality or morbidity. The discharge disposition of patients to home or rehabilitation were same regardless of the patient underwent TAC or partial colectomy.

Implications. If the disease pathology limited to one area of the colon, partial colectomy can be an alternative procedure for the FCDC patients.

## Data Availability

Data are available from the American College of Surgeons.

## Abbreviations

FCDC: Fulminant Clostridium Difficile Colitis
TAC: The Total Abdominal Colectomy
NSQIP: The National Surgical Quality Improvement Program
LOS: Length of stay
CI: Confidence interval
LI: Loop ileostomy
PCR: Polymerase chain reaction
EIA: Enzyme immunoassays
ASA: American Society of Anesthesiologists
DM: Diabetes mellitus
COPD: Chronic obstructive pulmonary disease
HTN: Hypertension on medication
CRF: Chronic renal failure
CRF-D: Chronic renal failure on dialysis
WSES: World Society of Emergency Surgery

## References

[1] Loo VG, Poirier L, Miller MA (2005). A predominantly clonal multi-institutional outbreak of Clostridium difficile-associated diarrhea with high morbidity and mortality. N Engl J Med.

[2] Dallal RM, Harbrecht BG, Boujoukas AJ, Sirio CA, Farkas LM, Lee KK, Simmons RL (2002). Fulminant *Clostridium difficile*: an underappreciated and increasing cause of death and complications. Ann Surg.

[3] Ahmed N, Kuo YH (2020). Early colectomy safes lives in toxic mega-colon due to *Clostridium difficile* infection. Southern Med J.

[4] Stewart D, Hollenbeak C, Wilson M (2013). Is colectomy for fulminant Clostridium difficile colitis life saving? A systematic review. Colorectal Dis.

[5] Napolitano LM, Edmiston CE (2017). Clostridium difficile disease: diagnosis, pathogenesis, and treatment update. Surgery.

[6] Neal M, Alverdy J, Hall D (2011). Diverting loop ileostomy and colonic lavage: an alternative to total abdominal colectomy for the treatment of severe, complicated *Clostridium difficile* infections. Ann Surg.

[7] Fashandi AZ, Martin AN, Wang PT, Hedrick TL, Friel CM, Smith PW, Hays RA, Hallowell PT (2017). An institutional comparison of total abdominal colectomy and diverting loop ileostomy and colonic lavage in the treatment of severe, complicated *Clostridium difficile* infections. Am J Surg.

[8] Peprah D, Chiu AS, Jean RA, Pei KY (2019). Comparison of outcomes between total abdominal and partial colectomy for the management of severe, complicated *Clostridium difficile* infection. J Am Coll Surg.

[9] Christl SU, Scheppach W (1997). Metabolic consequences of total colectomy. Scand J Gastroenterol Suppl.

[10] Austin PC (2011). An introduction to propensity score methods for reducing the effects of confounding in observational studies. Multivar Behav Res.

[11] https://www.facs.org/quality-programs/acs-nsqip, Access date September 3, 2020

[12] Ho DE, Imai K, King G, Stuart EA (2011). MatchIt: nonparametric preprocessing for parametric causal inference. J Stat Softw.

[13] R Core Team (2020). R: A language and environment for statistical computing. R Foundation for Statistical Computing, Vienna, Austria. https://www.R-project.org/.

[14] Sailhamer EA, Carson K, Chang Y (2009). Fulminant *Clostridium difficile* colitis: patterns of care and predictors of mortality. Arch Surg.

[15] Sartelli M, Malangoni MA, Abu-Zidan FM (2015). WSES guidelines for management of *Clostridium difficile* infection in surgical patients. World J Emerg Surg.

[16] Sartelli M, Di Bella S, McFarland LV (2019). 2019 update of the WSES guidelines for management of Clostridioides (Clostridium) difficile infection in surgical patients. World J Emerg Surg.

[17] Koss A, Clark MA, Sanders A, Morton D, Keighley MR, Goh J (2005). The outcome of surgery in fulminant *Clostridium difficile* colitis. Colorectal Dis.

[18] Byrn JC, Maun DC, Gingold DS, Baril DT, Ozao JJ, Divino CM (2008). Predictors of mortality after colectomy for fulminant *Clostridium difficile* Colitis. Arch Surg.

[19] Gillies MA, Harrison EM, Pearse RM, Garrioch S, Haddow C, Smyth L, Parks R, Walsh TS, Lone NI (2017). Intensive care utilization and outcomes after high-risk surgery in Scotland: a population-based cohort study. Br J Anaesth.

[20] Payá-Llorente C, Martínez-López E, Sebastián-Tomás JC (2020). The impact of age and comorbidity on the postoperative outcomes after emergency surgical management of complicated intra-abdominal infections. Sci Rep.

